# [Sulfonyl­bis­(bromo­methyl­ene)]di­benzene

**DOI:** 10.1107/S2414314621013511

**Published:** 2022-01-07

**Authors:** Peter W. R. Corfield

**Affiliations:** a Department of Chemistry, Fordham University, 441 East Fordham Road, Bronx, NY 10458, USA; Vienna University of Technology, Austria

**Keywords:** crystal structure, diasteromer, sulfone, 1,3 elimination

## Abstract

The title compound, C_14_H_12_Br_2_O_2_S, crystallizes as the *meso* isomer of a diastereoisomeric pair. This structure determination was key to determining that the 1,3 elimination of bromine by tri­phenyl­phosphine occurs with inversion of the configuration at each of the two chiral carbon atoms. In the crystal, the molecules are linked by weak C—H⋯O and C—H⋯Br hydrogen bonds.

## Abstract 2

The title compound crystallizes as the mesomeric isomer, which helped establish that the 1,3 elimination of bromine by tri­phenyl­phosphine occurs with inversion of configuration at each chiral carbon atom.

## Graphical abstract

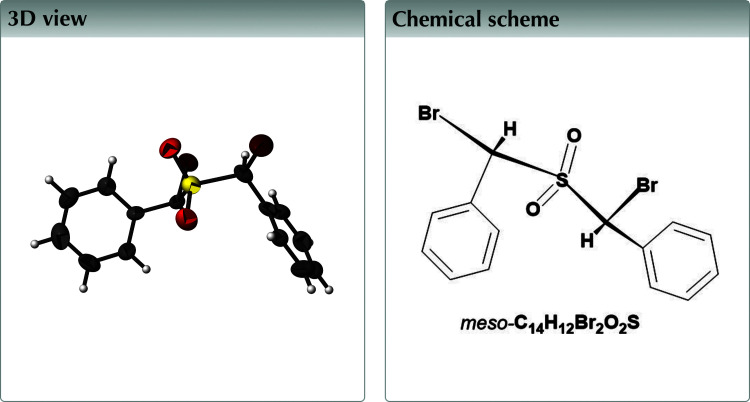

## Structure description

**Figure 1:**
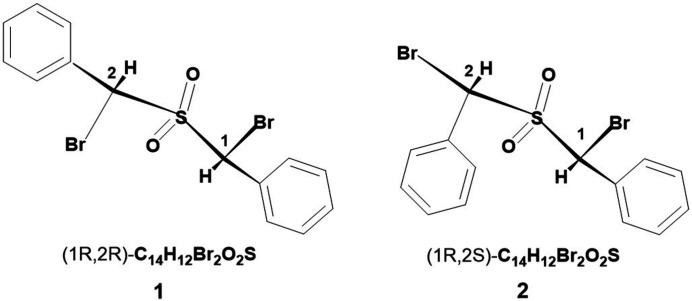
The two diastereoisomers, **1** and **2**, of PhCHBr·SO_2_·CHBrPh.

This structure determination was undertaken because of the high inter­est in the stereochemistry of 1,3 elimination reactions, particularly in the formation of α-sulfonyl carbanions (Cram *et al.*, 1966 ▸; Bordwell *et al.*, 1968*a* ▸). Two diastereoisomers, **1** and **2**, of PhCHBr·SO_2_·CHBrPh (Fig. 1 ▸) react stereospecifically with tri­phenyl­phosphine leading to 1,3 elimination of bromine followed by loss of sulfur dioxide to give stilbene, PhCH=CHPh, with **1** giving almost exclusively *trans* stilbene and **2** giving *cis* stilbene. Determination that the title compound **2** was the *meso* isomer was key to showing that the elimination occurred with double inversion of chirality at the C atoms (Bordwell *et al.*, 1968*b* ▸).

**Figure 2:**
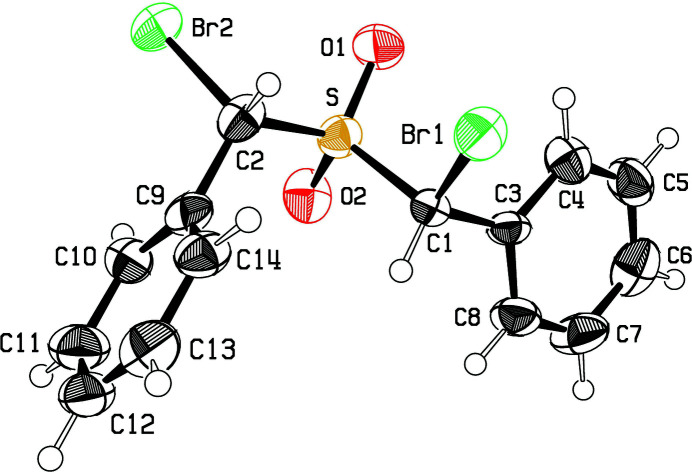
View of the title mol­ecule showing the atomic numbering and displacement ellipsoids at the 50% probability level.

Bond lengths and angles in the mol­ecular structure of **2** appear normal. As can be seen in Fig. 2 ▸, the chirality at C1 is *R* while that at C9 is *S*, indicating that this compound is the *meso* isomer. All mol­ecules in this centrosymmetric crystal will be the same *meso* isomer, although of course half will have opposite chiralities at C1 and C9. The C1—Br1 entity is *gauche* with respect to S—C2, whereas C2—Br2 is *trans* to S—C1, with conformational angles of −58.3 (5) and 171.3 (4)°, respectively.

**Figure 3:**
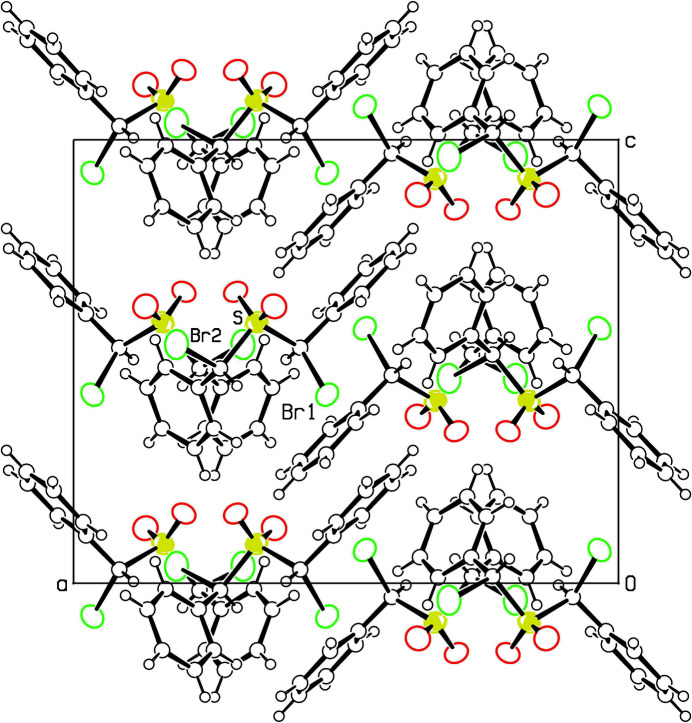
Projection of the crystal structure of **2** down the *b* axis. An arbitrary sphere size is given for C and H atoms, and a 50% probability level for the displacement ellipsoids of Br, S and O atoms. The reference mol­ecule has Br and S atoms identified.

The packing diagram (Fig. 3 ▸) shows the sulfone O atoms and the Br atoms projecting into hydro­phobic areas of the crystal. A number of putative C—H⋯O and C—H⋯Br inter­molecular hydrogen-bonding contacts are given in Table 1 ▸. The C⋯O distances range from 3.46 (2) to 3.55 (2) Å while angles at the H atom are in the general range of 120–130°. The three C⋯Br distances listed are longer, with a range of 3.74 (2) to 3.79 (2) Å and there is more variation in the angles at the H atoms. Inter­molecular H⋯H contacts are all greater than 2.5 Å except for H6⋯H10(*x* − 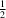 , *y*, 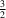 − *z*), which is 2.36 Å.

## Synthesis and crystallization

Details of the synthesis of the title compound are not given in the Bordwell papers, but details of two methods of preparing the compound are given in Carpino *et al.* (1971 ▸).

## Refinement

Crystal data, data collection and structure refinement details are summarized in Table 2 ▸.

In 1967, when this dataset was collected, mechanical failures were frequent enough that minimum redundancy was sought. This accounts for the low resolution of the data and the lack of symmetry-equivalents. An empirical absorption correction involving a 24-parameter fit was made with *XABS2* (Parkin *et al.*, 1995 ▸), which led to a much smoother difference-Fourier map. The H atoms attached to chiral C1 and C2 atoms were located as the two highest peaks on a difference map calculated without their contributions.

In the final refinements, the phenyl ring carbon atoms were refined as rigid groups in order to keep a reasonable ratio of observations to refined parameters. The C—C distance in the phenyl rings was set at 1.372 Å to minimize the weighted *R* factor. Although this distance is a little less than the average 1.39 Å usually found, a number of well-refined sulfone structures in the Cambridge Structural Database (Groom *et al.*, 2016 ▸) have C—C distances less than 1.39 Å, see: TUXFIC02 (Eccles *et al.*, 2011 ▸), BECRAE (Malwal & Chakrapani, 2015 ▸), GIPQON (Periasamy *et al.*, 2013 ▸), HEXLOO (Matsumoto *et al.*, 2018 ▸). The phenyl and H atoms attached to chiral C atoms all were constrained to lie in their expected positions, with C—H distances of 0.93 and 0.98 Å respectively, and displacement parameters set at 1.2*U*_eq_ for the adjoining carbon atoms.

## Supplementary Material

Crystal structure: contains datablock(s) I. DOI: 10.1107/S2414314621013511/wm4157sup1.cif

Structure factors: contains datablock(s) I. DOI: 10.1107/S2414314621013511/wm4157Isup2.hkl

Supporting information file. DOI: 10.1107/S2414314621013511/wm4157Isup3.cml

CCDC reference: 2130377

Additional supporting information:  crystallographic information; 3D view; checkCIF report

## Figures and Tables

**Table 1:** Hydrogen-bond geometry (Å, °)

*D*—H⋯*A*	*D*—H	H⋯*A*	*D*⋯*A*	*D*—H⋯*A*
C6—H6⋯O1^i^	0.93	2.92	3.523 (14)	123
C7—H7⋯O1^i^	0.93	2.80	3.462 (13)	129
C11—H11⋯O1^ii^	0.93	2.92	3.486 (14)	120
C12—H12⋯O2^iii^	0.93	2.89	3.545 (15)	128
C14—H14⋯O1^iv^	0.93	2.86	3.539 (17)	131
C14—H14⋯O2^iv^	0.93	2.86	3.548 (14)	132
C7—H7⋯Br1^v^	0.93	3.18	3.777 (14)	124
C8—H8⋯Br2^ii^	0.93	2.88	3.789 (15)	166
C13—H13⋯Br2^iv^	0.93	3.12	3.741 (19)	126

Symmetry codes: (i) 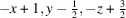 ; (ii) 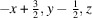 ; (iii) 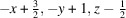 ; (iv) 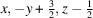 ; (v) 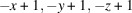 .

**Table 2:** Experimental details

Crystal data	
Chemical formula	C_14_H_12_Br_2_O_2_S
*M*_r_	404.12
Crystal system, space group	Orthorhombic, *P* *b* *c* *a*
Temperature (K)	295
*a*, *b*, *c* (Å)	16.53 (10), 12.81 (5), 13.46 (7)
*V* (Å^3^)	2850 (25)
*Z*	8
Radiation type	Mo *K*α
μ (mm^−1^)	5.83
Crystal size (mm)	0.60 × 0.50 × 0.40

Data collection	
Diffractometer	Picker, punched card control
Absorption correction	Empirical (using intensity measurements) four-dimensional tensor analysis (Parkin *et al.*, 1995 ▸)
*T*_min_, *T*_max_	0.148, 0.226
No. of measured, independent and observed [*I* > 2σ(*I*)] reflections	1334, 1334, 1059
*R*_int_	0
θ_max_ (°)	20.0
(sin θ/λ)_max_ (Å^−1^)	0.482

Refinement	
*R*[*F* ^2^ > 2σ(*F* ^2^)], *wR*(*F* ^2^), *S*	0.046, 0.105, 1.10
No. of reflections	1334
No. of parameters	148
H-atom treatment	H-atom parameters constrained
Δρ_max_, Δρ_min_ (e Å^−3^)	0.33, −0.42

Computer programs: *PICK* (local program by J. A. Ibers), *PICKOUT* (local program by R. J. Doedens), *FORDAP* (local version), *SHELXL* (Sheldrick, 2015 ▸), *ORTEP*-III (Burnett & Johnson, 1996 ▸), *ORTEP-3 for Windows* (Farrugia, 2012 ▸), and *publCIF* (Westrip, 2010 ▸).

## full crystallographic data

### Crystal data

**Table:**

C_14_H_12_Br_2_O_2_S	*D*_x_ = 1.884 Mg m^−^^3^*D*_m_ = 1.86 (1) Mg m^−^^3^*D*_m_ measured by flotation in CH_3_I/CCl_4_
*M**_r_* = 404.12	Mo *K*α radiation, λ = 0.7107 Å
Orthorhombic, *P**b**c**a*	Cell parameters from 10 reflections
*a* = 16.53 (10) Å	θ = 3.1–16.1°
*b* = 12.81 (5) Å	µ = 5.83 mm^−^^1^
*c* = 13.46 (7) Å	*T* = 295 K
*V* = 2850 (25) Å^3^	Block, colorless
*Z* = 8	0.60 × 0.50 × 0.40 mm
*F*(000) = 1584	

### Data collection

**Table:**

Picker, punched card control diffractometer	*R*_int_ = 0
Radiation source: sealed X-ray tube	θ_max_ = 20.0°, θ_min_ = 2.5°
θ/2θ scans	*h* = 0→15
Absorption correction: empirical (using intensity measurements) four-dimensional tensor analysis (Parkin *et al.*, 1995)	*k* = 0→12
*T*_min_ = 0.148, *T*_max_ = 0.226	*l* = 0→12
1334 measured reflections	3 standard reflections every 200 reflections
1334 independent reflections	intensity decay: 0(1)
1059 reflections with *I* > 2σ(*I*)	

### Refinement

**Table:**

Refinement on *F*^2^	Primary atom site location: heavy-atom method
Least-squares matrix: full	Secondary atom site location: difference Fourier map
*R*[*F*^2^ > 2σ(*F*^2^)] = 0.046	Hydrogen site location: mixed
*wR*(*F*^2^) = 0.105	H-atom parameters constrained
*S* = 1.10	*w* = 1/[σ^2^(*F*_o_^2^)] where *P* = (*F*_o_^2^ + 2*F*_c_^2^)/3
1334 reflections	(Δ/σ)_max_ < 0.001
148 parameters	Δρ_max_ = 0.33 e Å^−^^3^
0 restraints	Δρ_min_ = −0.42 e Å^−^^3^

### Special details

**Table:**

Geometry. All esds (except the esd in the dihedral angle between two l.s. planes) are estimated using the full covariance matrix. The cell esds are taken into account individually in the estimation of esds in distances, angles and torsion angles; correlations between esds in cell parameters are only used when they are defined by crystal symmetry. An approximate (isotropic) treatment of cell esds is used for estimating esds involving l.s. planes.
Refinement. After the empirical absorption correction with XABS2, a difference map based upon all of the atoms except H1 and H2 clearly revealed H1 and H2 as the two highest peaks.

### Fractional atomic coordinates and isotropic or equivalent isotropic displacement parameters (Å^2^)

**Table:**

	*x*	*y*	*z*	*U*_iso_*/*U*_eq_	
Br1	0.53460 (5)	0.75393 (8)	0.42515 (7)	0.0479 (4)	
Br2	0.81089 (6)	0.86936 (8)	0.53831 (8)	0.0592 (4)	
S	0.66395 (13)	0.74518 (19)	0.58854 (17)	0.0397 (7)	
O1	0.6327 (4)	0.8427 (5)	0.6245 (5)	0.0514 (18)	
O2	0.7024 (4)	0.6746 (5)	0.6565 (4)	0.0522 (18)	
C1	0.5828 (5)	0.6707 (6)	0.5315 (6)	0.035 (2)	
H1	0.605758	0.607183	0.502400	0.042*	
C2	0.7319 (5)	0.7715 (6)	0.4857 (6)	0.039 (2)	
H2	0.701115	0.807103	0.433486	0.047*	
C3	0.5219 (3)	0.6398 (5)	0.6091 (4)	0.034 (2)	
C4	0.4714 (4)	0.7116 (4)	0.6524 (5)	0.046 (3)	
H4	0.474013	0.781339	0.633557	0.055*	
C5	0.4171 (4)	0.6804 (6)	0.7235 (5)	0.055 (3)	
H5	0.382825	0.729053	0.752842	0.066*	
C6	0.4132 (3)	0.5774 (7)	0.7513 (4)	0.062 (3)	
H6	0.376382	0.556327	0.799451	0.074*	
C7	0.4637 (4)	0.5057 (4)	0.7079 (5)	0.062 (3)	
H7	0.461127	0.435885	0.726776	0.075*	
C8	0.5181 (4)	0.5368 (5)	0.6369 (5)	0.048 (3)	
H8	0.552316	0.488169	0.607490	0.058*	
C9	0.7698 (4)	0.6757 (4)	0.4412 (5)	0.044 (3)	
C10	0.8307 (4)	0.6217 (5)	0.4877 (4)	0.041 (2)	
H10	0.848985	0.643029	0.549794	0.050*	
C11	0.8646 (3)	0.5363 (5)	0.4425 (6)	0.059 (3)	
H11	0.905920	0.499671	0.473964	0.071*	
C12	0.8377 (4)	0.5049 (4)	0.3508 (6)	0.057 (3)	
H12	0.860697	0.446934	0.320211	0.069*	
C13	0.7768 (4)	0.5589 (6)	0.3044 (4)	0.054 (3)	
H13	0.758539	0.537555	0.242287	0.064*	
C14	0.7429 (3)	0.6443 (5)	0.3496 (5)	0.043 (3)	
H14	0.701603	0.680914	0.318116	0.052*	

### Atomic displacement parameters (Å^2^)

**Table:**

	*U*^11^	*U*^22^	*U*^33^	*U*^12^	*U*^13^	*U*^23^
Br1	0.0465 (6)	0.0488 (7)	0.0482 (6)	−0.0038 (5)	−0.0090 (5)	0.0088 (5)
Br2	0.0507 (7)	0.0452 (7)	0.0817 (8)	−0.0152 (5)	−0.0008 (6)	−0.0116 (6)
S	0.0405 (14)	0.0389 (15)	0.0397 (15)	−0.0038 (13)	−0.0045 (12)	−0.0029 (13)
O1	0.056 (4)	0.043 (4)	0.055 (4)	0.001 (3)	0.000 (3)	−0.018 (4)
O2	0.049 (4)	0.066 (4)	0.042 (4)	0.005 (4)	−0.010 (3)	0.014 (4)
C1	0.032 (5)	0.027 (5)	0.046 (6)	0.002 (4)	0.005 (5)	−0.001 (5)
C2	0.040 (6)	0.042 (7)	0.035 (5)	−0.009 (5)	−0.007 (5)	0.011 (5)
C3	0.040 (6)	0.024 (6)	0.038 (6)	0.000 (5)	−0.004 (5)	−0.005 (5)
C4	0.047 (7)	0.050 (7)	0.041 (6)	0.006 (6)	−0.001 (5)	0.006 (5)
C5	0.049 (7)	0.057 (8)	0.058 (8)	0.010 (6)	0.001 (6)	−0.016 (6)
C6	0.056 (8)	0.086 (9)	0.043 (6)	−0.019 (7)	0.004 (6)	−0.014 (7)
C7	0.080 (8)	0.037 (7)	0.070 (8)	−0.015 (7)	0.011 (6)	0.006 (6)
C8	0.058 (8)	0.035 (7)	0.052 (7)	0.000 (5)	0.014 (6)	−0.011 (5)
C9	0.043 (6)	0.031 (6)	0.056 (8)	−0.005 (5)	0.003 (5)	0.015 (6)
C10	0.049 (7)	0.039 (6)	0.036 (6)	0.003 (5)	0.007 (5)	0.006 (6)
C11	0.067 (8)	0.037 (7)	0.073 (9)	0.004 (6)	−0.004 (7)	0.002 (6)
C12	0.068 (8)	0.034 (6)	0.069 (9)	−0.007 (6)	0.027 (6)	−0.005 (6)
C13	0.063 (8)	0.053 (8)	0.046 (7)	−0.011 (6)	0.006 (6)	−0.004 (6)
C14	0.049 (6)	0.034 (7)	0.047 (7)	−0.002 (5)	−0.009 (6)	0.008 (5)

### Geometric parameters (Å, º)

**Table:**

Br1—C1	1.954 (10)	C6—C7	1.3720
Br2—C2	1.943 (10)	C6—H6	0.9300
S—O2	1.435 (7)	C7—C8	1.3720
S—O1	1.436 (7)	C7—H7	0.9300
S—C1	1.817 (10)	C8—H8	0.9300
S—C2	1.815 (11)	C9—C10	1.3720
C1—C3	1.503 (11)	C9—C14	1.3720
C1—H1	0.9800	C10—C11	1.3720
C2—C9	1.502 (10)	C10—H10	0.9300
C2—H2	0.9800	C11—C12	1.3720
C3—C4	1.3720	C11—H11	0.9300
C3—C8	1.3720	C12—C13	1.3720
C4—C5	1.3720	C12—H12	0.9300
C4—H4	0.9300	C13—C14	1.3720
C5—C6	1.3720	C13—H13	0.9300
C5—H5	0.9300	C14—H14	0.9300
			
O2—S—O1	119.5 (5)	C7—C6—C5	120.0
O2—S—C1	105.4 (5)	C7—C6—H6	120.0
O1—S—C1	109.5 (5)	C5—C6—H6	120.0
O2—S—C2	109.2 (5)	C6—C7—C8	120.0
O1—S—C2	108.5 (4)	C6—C7—H7	120.0
C1—S—C2	103.4 (5)	C8—C7—H7	120.0
C3—C1—S	109.8 (7)	C7—C8—C3	120.0
C3—C1—Br1	112.4 (6)	C7—C8—H8	120.0
S—C1—Br1	108.9 (5)	C3—C8—H8	120.0
C3—C1—H1	108.6	C10—C9—C14	120.0
S—C1—H1	108.6	C10—C9—C2	122.4 (6)
Br1—C1—H1	108.6	C14—C9—C2	117.5 (6)
C9—C2—S	114.2 (5)	C11—C10—C9	120.0
C9—C2—Br2	113.1 (6)	C11—C10—H10	120.0
S—C2—Br2	104.9 (5)	C9—C10—H10	120.0
C9—C2—H2	108.1	C10—C11—C12	120.0
S—C2—H2	108.1	C10—C11—H11	120.0
Br2—C2—H2	108.1	C12—C11—H11	120.0
C4—C3—C8	120.0	C13—C12—C11	120.0
C4—C3—C1	121.7 (6)	C13—C12—H12	120.0
C8—C3—C1	118.3 (6)	C11—C12—H12	120.0
C5—C4—C3	120.0	C12—C13—C14	120.0
C5—C4—H4	120.0	C12—C13—H13	120.0
C3—C4—H4	120.0	C14—C13—H13	120.0
C4—C5—C6	120.0	C13—C14—C9	120.0
C4—C5—H5	120.0	C13—C14—H14	120.0
C6—C5—H5	120.0	C9—C14—H14	120.0
			
O2—S—C1—C3	63.6 (6)	C3—C4—C5—C6	0.0
O1—S—C1—C3	−66.2 (6)	C4—C5—C6—C7	0.0
C2—S—C1—C3	178.3 (5)	C5—C6—C7—C8	0.0
O2—S—C1—Br1	−172.9 (4)	C6—C7—C8—C3	0.0
O1—S—C1—Br1	57.3 (5)	C4—C3—C8—C7	0.0
C2—S—C1—Br1	−58.3 (5)	C1—C3—C8—C7	179.5 (6)
O2—S—C2—C9	47.6 (7)	S—C2—C9—C10	−74.4 (7)
O1—S—C2—C9	179.5 (6)	Br2—C2—C9—C10	45.5 (7)
C1—S—C2—C9	−64.3 (7)	S—C2—C9—C14	107.3 (6)
O2—S—C2—Br2	−76.8 (6)	Br2—C2—C9—C14	−132.8 (4)
O1—S—C2—Br2	55.1 (5)	C14—C9—C10—C11	0.0
C1—S—C2—Br2	171.3 (4)	C2—C9—C10—C11	−178.2 (6)
S—C1—C3—C4	71.0 (6)	C9—C10—C11—C12	0.0
Br1—C1—C3—C4	−50.4 (7)	C10—C11—C12—C13	0.0
S—C1—C3—C8	−108.5 (5)	C11—C12—C13—C14	0.0
Br1—C1—C3—C8	130.1 (4)	C12—C13—C14—C9	0.0
C8—C3—C4—C5	0.0	C10—C9—C14—C13	0.0
C1—C3—C4—C5	−179.5 (6)	C2—C9—C14—C13	178.3 (5)

### Hydrogen-bond geometry (Å, º)

**Table:**

*D*—H···*A*	*D*—H	H···*A*	*D*···*A*	*D*—H···*A*
C6—H6···O1^i^	0.93	2.92	3.523 (14)	123
C7—H7···O1^i^	0.93	2.80	3.462 (13)	129
C11—H11···O1^ii^	0.93	2.92	3.486 (14)	120
C12—H12···O2^iii^	0.93	2.89	3.545 (15)	128
C14—H14···O1^iv^	0.93	2.86	3.539 (17)	131
C14—H14···O2^iv^	0.93	2.86	3.548 (14)	132
C7—H7···Br1^v^	0.93	3.18	3.777 (14)	124
C8—H8···Br2^ii^	0.93	2.88	3.789 (15)	166
C13—H13···Br2^iv^	0.93	3.12	3.741 (19)	126

Symmetry codes: (i) −*x*+1, *y*−1/2, −*z*+3/2; (ii) −*x*+3/2, *y*−1/2, *z*; (iii) −*x*+3/2, −*y*+1, *z*−1/2; (iv) *x*, −*y*+3/2, *z*−1/2; (v) −*x*+1, −*y*+1, −*z*+1.
